# The Antibacterial and Remineralization Effect of Silver-Containing Mesoporous Bioactive Glass Sealing and Er-YAG Laser on Dentinal Tubules Treated in a *Streptococcus mutans* Cultivated Environment

**DOI:** 10.3390/ph14111124

**Published:** 2021-11-04

**Authors:** Jung-Chang Kung, Wei-Hsun Wang, Yu-Ching Chiang, Yuan-Ting Yang-Wang, Yueh-Ching Wang, Wen-Cheng Chen, Chi-Jen Shih

**Affiliations:** 1 School of Dentistry, College of Dental Medicine, Kaohsiung Medical University, Kaohsiung 807, Taiwan; kung1129@kmu.edu.tw; 2 Department of Dentistry, Division of Family Dentistry, Kaohsiung Medical University Hospital, Kaohsiung 807, Taiwan; 3 Department of Dentistry, Kaohsiung Municipal Ta-Tung Hospital, Kaohsiung 801, Taiwan; 4 Department of Orthopedic Surgery, Changhua Christian Hospital, Changhua 500, Taiwan; wangweihsun@gmail.com; 5 School of Medicine, College of Medicine, Kaohsiung Medical University, Kaohsiung 807, Taiwan; 6 Department of Medical Imaging and Radiology, Shu-Zen Junior College of Medicine and Management, Kaohsiung 821, Taiwan; 7 College of Medicine, National Chung Hsing University, Taichung 402, Taiwan; 8 Department of Chemical Engineering, National United University, Miaoli 360, Taiwan; 9 Department of Golden-Ager Industry Management, Chaoyang University of Technology, Taichung 413, Taiwan; 10 Department of Fragrance and Cosmetic Science, College of Pharmacy, Kaohsiung Medical University, Kaohsiung 807, Taiwan; qpy261234@gmail.com (Y.-C.C.); kmu102012062@gmail.com (Y.-T.Y.-W.); yuehchingw@gmail.com (Y.-C.W.); 11 Advanced Medical Devices and Composites Laboratory, Department of Fiber and Composite Materials, Feng Chia University, Taichung 402, Taiwan; 12 Department of Medical Research, Kaohsiung Medical University Hospital, Kaohsiung 807, Taiwan

**Keywords:** antibacterial, remineralization, Er-YAG laser, dentinal tubules, *Streptococcus mutans*, dentin hypersensitivity

## Abstract

The aim of this study was to evaluate the remineralization and antibacterial effect of silver-containing mesoporous bioactive glass (MBG-Ag) sealing combined with Er:YAG laser irradiation on human demineralized dentin specimens in a *Streptococcus mutans* cultivated environment. A total of 48 human dentin specimens were randomly divided into four groups. The characteristics of MBG-Ag and the occlusion efficiency of the dentinal tubules were analyzed using X-ray diffraction patterns, Fourier-transform infrared spectroscopy, scanning electron microscope images and energy dispersive X-ray spectroscopy. Moreover, the antibacterial activity against *Streptococcus mutans* was evaluated by colony formation assay. The results showed that the dentin specimens with Er:YAG laser irradiation can form a melted occlusion with a size of 3–4 µm. MBG-Ag promoted the deposition of numerous crystal particles on the dentinal surface, reaching the deepest penetration depth of 70 μm. The results suggested that both MBG-Ag and laser have the ability to enhance the remineralization and precipitation of hydroxyapatite crystals. While the results showed that MBG-Ag sealing combined with the thermomechanical subablation mode of Er:YAG laser irradiation-induced dense crystalline deposition, reaching a penetration depth of more than 300 µm, silver nanoparticles without good absorption of the Er:YAG laser resulted in a heterogeneous radiated surface. Er:YAG laser irradiation with a low energy and pulse rate cannot completely inhibit the growth of *S. mutans*, but MBG-Ag sealing reached the bactericidal concentration. It was concluded that the simultaneous application of MBG-Ag sealing and Er:YAG laser treatment can prevent the drawbacks of their independent uses, resulting in a superior form of treatment for dentin hypersensitivity.

## 1. Introduction

The symptom of dentin hypersensitivity is mainly caused by dentinal tubule exposure resulting from enamel damage, erosion, abrasion, aging and gingival recession. The most widely accepted mechanism is hydrodynamic theory [1,2]. Fluids within the dentinal tubules are affected by changes in temperature or osmotic pressure, which indirectly leads to pain. The factors that cause this pain can be attributed to chemical factors, such as bacterial erosion; physical factors, such as improper brushing method; bite force factors, such as occlusal wear; and pathological factors, such as gingival recession [3]. The occlusion of dentinal tubules caused by chemical factors, such as beverages and acidogenic bacteria that cause a continuously acidic environment in the mouth, is notable [4,5,6]. The dissolution of hydroxyapatite induced by abrasion leads to the expansion of the inner diameter of dentinal tubules and the exposure of long collagen fibrils [7]. The main pathogenic bacteria is *Streptococcus mutans* (*S. mutans*), which decomposes food residue in the oral cavity and causes an acidic environment. It can easily adhere to the cracks and grooves of the teeth and metabolize carbohydrates in the oral cavity. Lactic acid is produced in the cavity, and then glucans is produced, leading to demineralization and acid erosion of the enamel [8]. In contrast, dietary food residues, bacteria and cell debris accumulate on the tooth surface through dextran mediation, gradually causing the formation of dental plaque [9,10]. Biofilms can be resistant to oral cleaning, deteriorate enamel demineralization, damage the tooth structure, cause caries [11], and ultimately, result in the exposure of dentinal tubules and dentin hypersensitivity. The diverse bacteria continue to grow inside dentinal tubules, and pulp cavities may lead to further infection and other types of oral diseases.

Nowadays, dentin hypersensitivity treatment is carried out with the use of a nerve desensitizer, dentin sealers, and laser treatment [2]. However, the nerve desensitizer requires long-term use to show results, and the effectiveness is lost when treatment is discontinued. Dentin sealers such as fluoride have a small amount of sealer and a shallow penetration depth, which are liable to fall off due to brushing-induced erosion. Treatment for long-term caries prevention is currently lacking.

The dental laser is a novel device that can be used to treat dentin hypersensitivity. The thermomechanical ablation mechanism of the laser can effectively reduce the diameter of dentinal tubules and decrease the movement of dentinal fluid [12]. Furthermore, the appearance, topography, stiffness and bacterial adhesion forces may impact the dentin surface through the photothermal and thermomechanical ablation mechanism [13,14,15]. However, the deep penetration depth and high-speed heating rate cause irreversible heat damage to the surrounding tissues and pulp cavity, which represent the development restrictions of the dental laser [7]. While conventional laser dental treatments face obstacles because of the absorption wavelength and the additional damage to the surrounding tissues, the Er:YAG laser has a specificity absorption wavelength of 2.94 μm for water and dentin, suggesting promising application in dental treatment for hard and soft tissues [16,17]. Not only is it safe due to the shallow energy penetration depth, but it is also capable of cleaning, removing caries and occluding dentinal tubules.

Mesoporous bioactive glass (MBG) is a material that is compatible with dentin. The crystal particles of MBG can release calcium ions and phosphorus ions to reduce demineralization and promote precipitation into the dentinal tubules [18]. The precipitated crystalline layer is similar to the dentin mineral hydroxyapatite, which can promote stronger adhesion of crystal particles to the dentin surface. AgNPs are well known for their broad-spectrum antibacterial effect at low concentrations, and the antibacterial activity of AgNPs is far greater than their cytotoxicity [19,20]. The release of silver ions affects the reactive side chain of bacterial collagenase, which inactivates the catalytic functions of bacteria and preserves dentin collagen [21]. Silver possesses the capability to inhibit the activity of matrix metalloproteinases (MMPs) [22]. It is presumed that Ag^+^ occupies the sites of Ca^2+^ in collagen fibrils and protects collagen from the degradation caused by MMPs through competitive inhibition [22]. AgNPs have shown the ability to regulate collagen deposition and direct proper collagen matrix arrangement in the research of wound healing [23]. Studies have suggested that silver is able to improve the chemical structure and stability of collagen in demineralized dentin [22]. Therefore, we synergized MBG-Ag as a sealer and used Er:YAG laser treatment human dentin specimens, and analyzed the characteristics and antibacterial activity of these specimens after MBG-Ag sealing and Er:YAG laser irradiation in this study.

## 2. Results

### 2.1. This X-ray Diffractometer (XRD) Analysis

**Figure 1:**
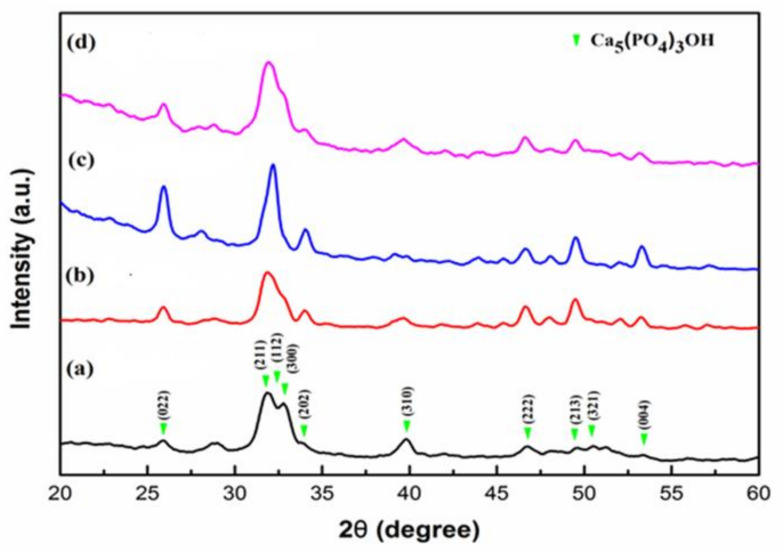
XRD results of the dentin specimens (**a**) without any treatment, (**b**) with Er:YAG laser treatment, (**c**) with MBG-Ag 40PA sealing and (**d**) with MBG-Ag 40PA sealing and Er:YAG laser treatment.

The results of XRD patterns are shown in Figure 1. The group consisted of the dentin specimens without any treatment, and its diffraction peaks were 25.87°, 31.7°, 32.1°, 32.9°, 34.04°, 39.8°, 46.71°, 49.46°, 50.49°, and 53.14°. These diffraction peaks were compared with JCPD 09–0432 and were observed to match the diffraction peaks of hydroxyapatite. Therefore, the composition of the dentin specimens was hydroxyapatite and had (002), (211), (112), (300), (202), (310), (222), (213), (321), and (004) crystal planes. Group b consisted of the dentin specimens that received Er:YAG laser treatment. Comparing the diffraction peaks of Groups b and a, it was observed that Group b had slightly lower peaks at 31.7° and sharper peaks at 32.1° than those of Group a. Group c consisted of the dentin specimens that received MBG-Ag 40PA sealing. Group d consisted of the dentin specimens that received MBG-Ag 40PA sealing and Er:YAG laser treatment. The diffraction peaks intensities of the Groups c and d were stronger than those of Group b. The results indicate that both MBG-Ag 40PA sealing and Er:YAG laser treatment had the ability to induce remineralization of the dentin specimens.

### 2.2. Fourier Transform Infrared (FTIR) Spectrometer Analysis

**Figure 2:**
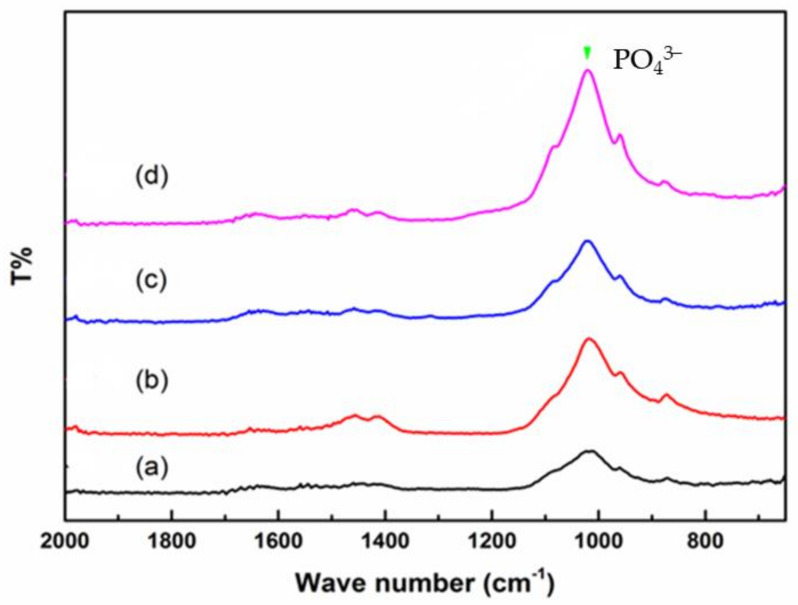
FTIR results of the dentin specimens (**a**) without any treatment, (**b**) with Er:YAG laser treatment, (**c**) with MBG-Ag 40PA sealing and (**d**) with MBG-Ag 40PA sealing and Er:YAG laser treatment.

The characteristics of the dentin specimens were analyzed using FTIR spectra on attenuated total reflection mode. The FTIR spectrum recorded the main frequency of the dentin range from 2000 cm^−1^ to 650 cm^−1^, as the results in Figure 2 show. Group a consisted of the dentin specimens without any treatment, and it showed a broad band at approximately 1000 cm^−1^, which was attributed to the presence of PO_4_^3−^ vibration [24]. The highest intensity of the peak revealed the formation of phosphate ions, which formed a hydroxyapatite Ca_10_(PO_4_)_5_(OH)_2_ crystal. The spectra of Groups b–d showed an increase in intensity at the absorption peaks of PO_4_^3−^ vibration. The results show that both MBG-Ag 40PA sealing and Er:YAG laser treatment can promote the formation of hydroxyapatite.

### 2.3. Scanning Electron Microscope (SEM) and Energy Dispersive X-ray (EDX) Spectrometer Analysis

The morphology of the dentin surface, occlusion efficiency and penetration depth of dentinal tubules were observed via SEM.

**Figure 3:**
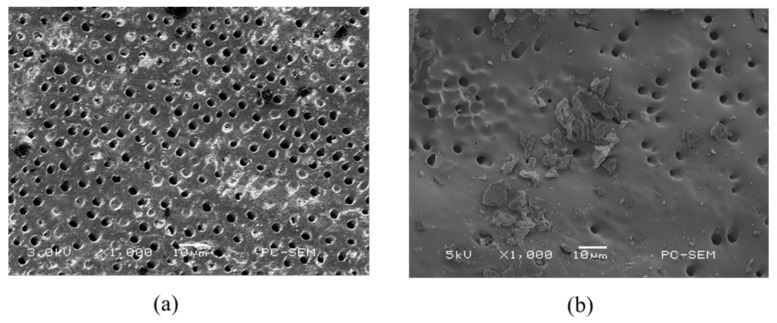

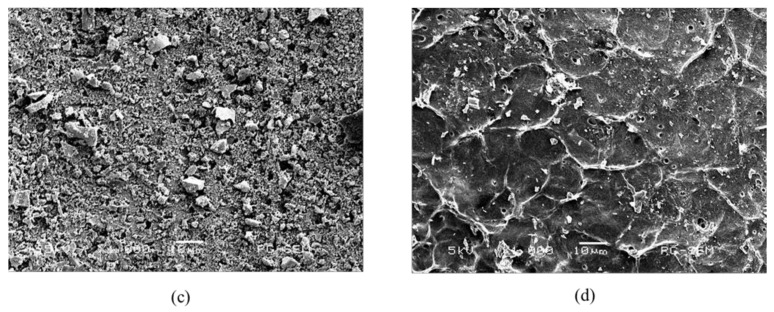
SEM images of the dentin specimens (**a**) without any treatment, (**b**) with Er:YAG laser treatment, (**c**) with MBG-Ag 40PA sealed and (**d**) with MBG-Ag 40PA sealing and Er:YAG laser treatment.

**Figure 4:**
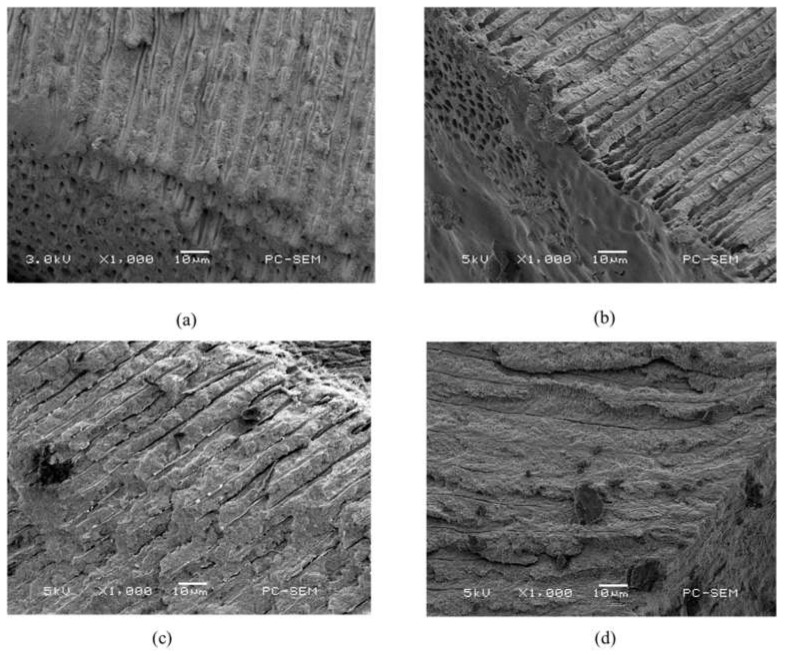
SEM images of cross-sections of the dentin specimens (**a**) without any treatment, (**b**) with Er:YAG laser treatment, (**c**) with MBG-Ag 40PA sealed and (**d**) with MBG-Ag 40PA sealing and Er:YAG laser treatment.

Figure 3a displays the SEM image of the dentin specimen that was not treated after removing the smear layer. It can be observed that the dentin pores were exposed. The diameter of dentin pores was about 2–5 um. Figure 3b shows the SEM image of the dentin specimen after Er:YAG laser treatment. It can be observed that a molten layer was formed on the surface of the dentin specimen. Some of the dentin pores were completely closed, and some of them were incompletely closed in a semi-melted form. Figure 3c shows the SEM image of the dentin specimen after MBG-Ag 40PA sealing. It can be observed that a large number of MBG-Ag crystal particles were sealed on the top of the dentin pores to achieve good occlusion efficiency. Figure 3d displays the SEM image of the dentin specimen after the combination of MBG-Ag 40PA sealing and Er:YAG laser treatment. The laser treatment caused the MBG-Ag to melt above the dentin pores. It can be observed that the bulging boundary and pores were connected by melting, thereby forming larger pores, and some pores were not completely closed. Comparing the SEM images presented in Figure 3b,d, it can be observed that the molten surface of the dentin specimen with only Er:YAG laser treatment was uniform and flat, without a bulging appearance. It was speculated that the Er:YAG laser was specific to the hydroxyapatite absorption peak. However, the nanosilvers in the MBG-Ag 40PA did not present good absorption of the Er:YAG laser, resulting in a bulging appearance. Figure 4 showed the cross-sectional SEM images of the dentin specimens of each group. The longitudinal cross-sectional view of Figure 4a shows that there was no precipitate inside the dentinal tubules. In Figure 4b, the dentin surface after Er:YAG laser treatment formed a melted and occluded surface that caused 63% of the dentinal tubule occlusion. However, some of the dentinal tubules were semi-closed and irregular in appearance, which resulted in the notable error bar of occlusion efficiency. The longitudinal section view was covered with smooth and uniform occlusion 3–4 µm in size, but some of the tubules were nearly exposed. Figure 4c shows that MBG-Ag formed numerous crystal particles on the surface of dentin and reached the deepest penetration depth of 68 µm. The occlusion efficiency was 94%. The longitudinal section view clearly shows that a great amount of materials penetrated the dentinal tubules. As the results in Figure 4d show, the dentin specimen after the combination of MBG-Ag 40PA sealing and Er:YAG laser treatment could form an occlusion on its surface. The occlusion efficiency was 96%. It reached a penetration depth of deeper than 300 µm. All of the statistical results of occlusion efficiency are summarized in Table 1.

**Figure 5:**
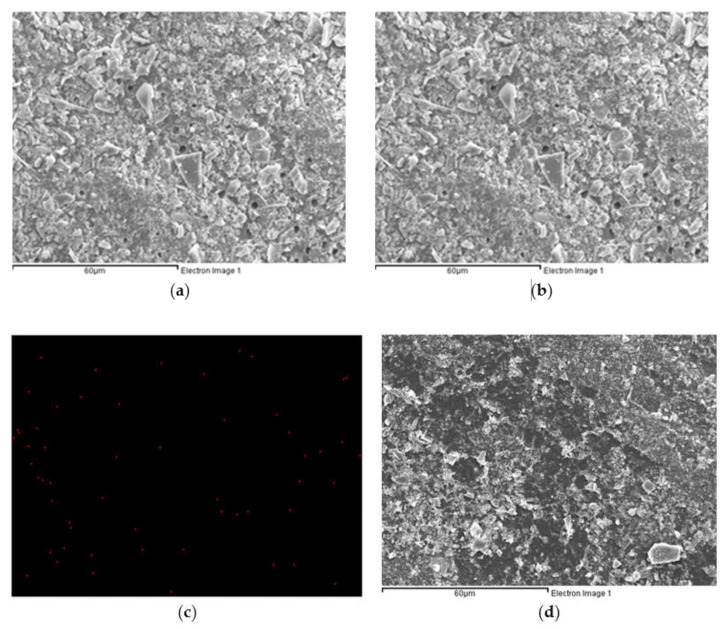
Dentin specimen after MBG-Ag 40PA sealing: (**a**) SEM image and (**b**) EDX Ag-mapping image; dentin specimen after MBG-Ag 40PA sealing and Er:YAG laser treatment: (**c**) SEM image and (**d**) EDX Ag-mapping image.

The SEM images and the EDX Ag-mapping images of dentin specimens after MBG-Ag 40PA sealing and MBG-Ag 40PA sealing combined with Er:YAG laser treatment are displayed in Figure 5. The distributed red dots are the positions where silver nanoparticles on dentin specimens were detected. In Figure 5b, it can be observed that the silver nanoparticles were uniformly dispersed on the dentin specimens, and the distribution area was wide and uniform. The EDX Ag-mapping image of the dentin specimen that received MBG-Ag 40PA sealing combined with Er:YAG laser treatment is shown in Figure 5d. The experimental results show that the distribution area of silver nanoparticles was significantly small. The result suggests that silver nanoparticles might penetrate dentinal tubules through the thermomechanical ablation mechanism of the laser and then indirectly cause a reduction in the detected content.

### 2.4. Antibacterial Activity Analysis

**Figure 6:**
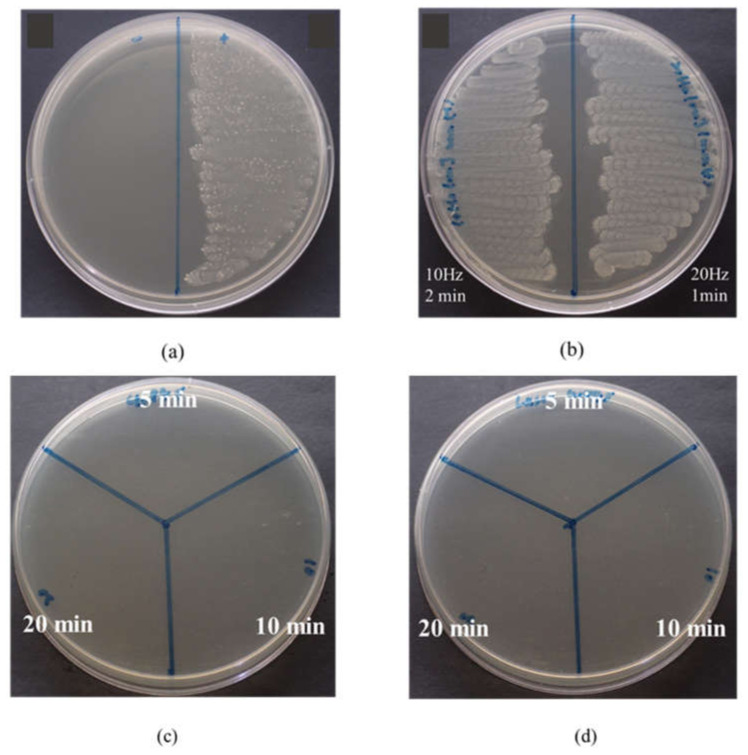
Results of colony-forming assay against *Streptococcus mutans* for (**a**) control groups, (**b**) Er:YAG laser treatment, (**c**) MBG-Ag 40PA sealing and (**d**) MBG-Ag 40PA sealing combined with Er:YAG laser treatment.

After sterilizing the dentin specimens from which the smear layer was removed, we simulated the bacterial infection of *Streptococcus mutans*, applied a minimum inhibitory concentration of 5 mg/mL, rinsed the specimens off with deionized water, cultured them in a liquid medium for 24 h, and then dipped them into a BHI agar with a cotton swab to test the colony-forming assay and observe its antibacterial activity. The antibacterial activity results of the colony formation assay are shown in Figure 6. The control group in Figure 6a (control (-) on the left side) confirms that the dentin specimen after disinfection and sterilization was in a clean and sterile state, and that it was not contaminated by bacteria after the sterilization was complete. The control (+) on the right side of the dentin specimen was obtained after simulating the infection of *Streptococcus mutans*. The growth of *Streptococcus mutans* on the BHI agar was dense and continuous, and its appearance was white. It was observed that the growth of the simulative *Streptococcus mutans* was normal. Figure 6b displays a group that received Er:YAG laser treatment (different time and frequency) after simulating *Streptococcus mutans* infection. It can be observed that the growth of bacteria on the BHI agar was the same as that of the control (+) on the right side of Figure 6a, and the bacteria were dense and continuous. Therefore, it could be inferred that using a laser alone could not completely and effectively inhibit the growth of bacteria. For the dentin specimens treated with MBG-Ag 40PA for 5, 10, and 20 min, which prevented the growth of *S. mutans*, no colonies formed on the BHI agar shown in Figure 6c. MBG-Ag 40PA sealing combined with Er:YAG Laser treatment is shown in Figure 6d. The results shown in Figure 6c,d are similar. The results indicate that MBG-Ag 40PA sealing allowed for the bactericidal concentration to be reached.

## 3. Discussion

In this study, the combination of bioactive glass and laser irradiation is proposed as an innovative form of treatment for dentin hypersensitivity. The mechanism of crystal precipitation suggests that bioactive glass applied to dentin initiates the condition of high concentrations of calcium ions and promotes the formation of calcium phosphate crystals. These crystals then precipitate on top of the dentin surface, and smaller crystals can penetrate the dentinal tubules [25]. Furthermore, mesoporous bioactive glass has the characteristics of high pore volume, high specific surface area, osteoinductivity and biocompatibility, which allow for the rapid release of a significant number of calcium ions [26,27]. However, crystal precipitation is not reactive with dentin, mesoporous bioactive glass can only block *Streptococcus mutans* and does not have antibacterial effects. It easily falls off due to daily acid abrasion and brush erosion [25]. Current studies have indicated that laser irradiation could effectively improve this reactivity and decrease the adhesion of bacteria. Our research team has published a study on the use of the sol-gel technique and evaporation-induced self-assembly (EISA) method to synthesize MBG-Ag with an ordered mesoporous structure and silver nanoparticles (AgNPs) encapsulated in it [28]. Although the antibacterial mechanisms of AgNPs have not been completely revealed, the main antibacterial mechanisms can be divided into three types: (1) destruction of the structure of the bacterial cell membrane, (2) inhibition of the formation of bacterial proteins and nucleic acids and (3) induction of the production of reactive oxygen species (ROS) [29]. Our research team has published another study that MBG-Ag with a powder-to-liquid ratio of 5 mg:1 mL was uniformly mixed with 40% phosphoric acid for 10 min to form MBG-Ag 40PA, then used it to seal dentin specimens. The experiment results found that a deeper penetration depth can be achieved in the dentin tubules and the possibility of erosion can be reduced [30]. 

In this study, MBG-Ag 40PA sealing and Er:YAG laser irradiation were applied to dentin specimens simulated by *Streptococcus mutans* to analyze their antibacterial and remineralization ability. Clinical research has indicated that the Er:YAG laser with a wavelength of 2.94 μm has a specific absorption wavelength for the main constituents of dental hard tissue. Compared with other lasers, it has a shallow energy penetration depth and results in less thermal damage to periodontal and pulp tissue. The dentin specimens treated with the Er:YAG laser formed occlusion with a size of 3–4 µm, but the homogeneity was clearly influenced by systematic error. Moreover, the explosive vaporization due to the thermomechanical ablation mechanism during dental treatment may still lead to irreversible damage of dental tissue and destruction of the hydroxyapatite structure [17]. The Er:YAG laser was applied with the lowest energy and frequency to develop a safe treatment strategy for patients in this study, and the SEM results show that the dentin surface was smooth under Er:YAG laser irradiation. As such, many researchers have suggested that this method, in addition to the thermal effect of the laser, successfully reduces the growth of bacteria. The roughness of the substratum surface was regarded as a significant factor influencing the adhesion of bacteria [14]. However, there remained a partially melted area on dentin surface treated with low-energy laser irradiation that suggested that the Er:YAG laser cannot have a bactericidal effect. On the contrary, MBG-Ag sealing combined with the thermal effect of laser irradiation promotes the evaporation of water in the interaction layer and forms a dense crystalline phase on dentin surface. This further suggests that this method improves the hardness and elastic modulus of the crystalline phase, enhances the resistance of abrasion challenge, improves the mechanical properties and reduces the collagen degradation of dentin [31]. The collagen of dentin as the scaffold for the growing of mineral crystals plays an essential role in dentin remineralization [21]. Moreover, the application of silver nanoparticles can regulate the deposition of collagen and promote further mineralization through the enhancement of collagen crosslinking [22]. The ability to inhibit the bacterial proteolysis can also allow for the degradation of dentin collagen to be avoided [32]. MBG-Ag sealing combined with laser irradiation can not only effectively improve the occlusion efficiency and penetration depth of dentinal tubules, but it also reduces the sensitivity caused by laser alone. The peaks of XRD patterns and FTIR spectra also demonstrated a significant increase. While the absorption of the Er:YAG laser by silver nanoparticles led to an uneven, occluded dentin surface, the penetration ability was not influenced. MBG-Ag 40PA penetrated the dentinal tubules via the photothermal mechanism, reducing the dentinal tubules’ permeability and reaching a penetration depth of more than 300 µm. It was estimated that a deeper penetration depth would help resist physical and chemical abrasion in the oral cavity, resulting in a longer-term anti-sensitivity effect. In addition, the results of the colony formation assay confirmed that MBG-Ag with a powder-to-liquid ratio of 5 mg:1 mL was mixed with 40% phosphoric acid uniformly and reached a bactericidal concentration. The strategy of using mesoporous bioactive glass containing silver nanoparticles combined with Er:YAG laser irradiation could treat dentin hypersensitivity and produce a long-lasting antibacterial adhesive effect on the surface of dentin.

## 4. Materials and Methods

### 4.1. Preparation of Materials

The silver-containing mesoporous bioactive glass (MBG-Ag) was synthesized using the sol–gel technique and the EISA method in this study. MBG-Ag belongs to the SiO_2_-CaO-P_2_O_5_-Ag system, in which the mole percentages of Si, Ca and P are 80, 15 and 5, respectively, and to which 1 mol% Ag was added. The raw materials included ethanol (99.5% purity) as a solvent; Pluronic F-127 (BASF Corp., Berlin, Germany) as a template surfactant; and tetraethyl orthosilicate (98.0% purity, ACROS Organics, Newark, NJ, USA), calcium nitrate tetrahydrate (98.5% purity, SHOWA, Kanagawa, Japan), triethyl phosphate (98.0% purity, FLUKA, Charlotte, NC, USA) and silver nitrate (99.8% purity, SHOWA, Kanagawa, Japan) as precursors. These raw materials were mixed to form a mixture. The mixture was continuously stirred at room temperature in the dark for 24 h, and then the macroporous template of polyurethane foam was soaked in the gel to age it for 10 min and dried at 100 °C for 24 h. The temperature of heat treatment was increased from room temperature to 600 °C at the heating rate of 10 °C/min, and the maximum temperature was maintained for 2 h to form MBG-Ag. MBG-Ag and 40% phosphoric acid (PA) were mixed uniformly at a powder-to-liquid ratio of 5 mg:1 mL to form MBG-Ag 40PA.

### 4.2. Preparation of Dentin Specimens

This study was authorized by the Institutional Review Board of Kaohsiung Medical University Memorial Hospital, Taiwan (KMUHIRB-E(I)-20180324, approved on 30 May 2019). Human premolars were collected from adults aged over 20 years old, and teeth with obvious defects, such as decay, stains, deformation, apical lesions and enamel hypoplasia, were excluded. Teeth with a complete structure were selected due to surgical or orthodontic reasons. Then, we separated the crown and cementoenamel junction of the teeth with high-speed diamond burs and cut 2–3 mm dentin sections with flat surfaces. The dentin specimens were disinfected with de-ionized water and 1.25% NaOCl. Then, we removed the smear layer on the surface with 40% phosphoric acid (PA) for 1 min to make a demineralized model [33]. 

### 4.3. Laser Treatment

An Er:YAG laser (SAPPHIRE, LIGHTMED Corp., San Clemente, CA, USA) emitting a wavelength of 2.94 μm and a pulse duration of 90 μs was applied to the surfaces of the dentin specimens in this study. The output energy was adjusted to 0.1 W, and a pulse frequency of 10 Hz was used with an angled handpiece to ensure optimal operation. The laser tip ST 100 was held vertical to the irradiated surface at a distance of 1 mm to avoid contact. The Er:YAG laser beam was operated with a sweeping motion and with energy of 10 mJ and a pulse frequency of 10 Hz to cover a 4 mm × 4 mm square for 60 s per specimen.

The dentin specimens were randomly allocated to four groups:

Group a: dentin specimens without treatment (control group);

Group b: dentin specimens with Er:YAG laser treatment;

Group c: dentin specimens with MBG-Ag 40PA sealing;

Group d: dentin specimens with MBG-Ag 40PA sealing and Er:YAG laser treatment.

### 4.4. Characteristics

The characteristics of dentin specimens after treatment were evaluated by X-ray diffractometry (XRD), Fourier-transform infrared (FTIR) spectrometry and scanning electron microscopy (SEM).

The X-ray diffraction patterns were recorded by XRD (XRD 6000, Shimadzu, Kyoto, Japan) with Cu Kα radiation (λ = 1.542 Å), voltage of 30 kV, current of 20 mA, scan range of 2θ = 20° – 60° and speed of 4°/min. The comparisons of crystal structures via Jade 6 XRD software were verified with a Joint Committee on Powder Diffraction Standards card (JCPDS). The absorbance peaks and bonds were assessed using FTIR spectrometry (Nicolet 6700, Thermo, Mundelein, IL, USA). The spectrum was recorded from 4000 cm^−1^ down to 650 cm^−1^ in attenuated total reflectance mode. The images of each group were recorded by SEM (JSM-6380, JEOL, Toyko, Japan) and field emission scanning electron microscopy (FESEM) (JSM-6330TF JEOL, Toyko, Japan). The qualitative analysis of Ag^+^ was carried out using energy dispersive X-ray (EDX) spectrometry (INCA x-stream, OXFORD, Concord, MA, USA). The specimens after pretreatment in groups were soaked in 2.5% glutaraldehyde solution for at least 1 h and rinsed with phosphate-buffered saline (PBS). Then, the specimens were sequentially dehydrated with ethanol from a low concentration (25%) to a high concentration (99.5%), dried and sputter-coated with gold. To observe the morphology and penetration depth of their profiles, the specimens were sectioned vertically. The appearance of the dentin specimens and the efficiency of penetration depth were simultaneously assessed by SEM. The developed formula was used for 4–5 SEM images, and the total number of dentinal tubules was divided by the number of recrystallized dentinal tubules (unit: μm). The penetration depth of tubule occlusion adopted the optimal parameter on SEM images [34].

### 4.5. Analysis of the Antibacterial Activity

The demineralized models of human dentin specimens were sterilized with 1.25% NaOCl and abundant de-ionized water to ensure the aseptic condition. The strain of *S. mutans* (American Type Culture Collection, ATCC 25175) was inoculated to 3–5 generations in brain heart infusion (BHI) broth and agar at 37 °C ± 2.5 °C for 24 h using the streaking method. The concentration of *S. mutans,* which was adjusted to the McFarland 0.5 scale (10^8^ CFU/mL) and diluted 10-fold to 10^6^ CFU/mL, was detected using a suspension turbidity detector (DEN-1, Biosan, Latvia, UK). Simulation of *S. mutans* infection was carried out for the dentin specimens treated with MBG-Ag 40PA sealing and Er:YAG laser, and they were inoculated in BHI broth at 37 °C ± 2.5 °C for 24 h. The antibacterial activity of each group was evaluated by colony formation assay, which was used to analyze the ability of *S. mutans* to form colonies on BHI agar.

### 4.6. Statistical Analysis

The experiments were repeated at least 3 times individually. The statistical results were analyzed using one-way ANOVA with Excel 2013. The differences in measurements were considered significant when *p* < 0.05.

## 5. Conclusions

Silver-containing mesoporous bioactive glass with a high specific surface area possesses antibacterial and remineralization abilities, which allows for crystal particles to penetrate the dentin surface and achieve an excellent occlusion rate. In the current study, silver nanoparticles were used to inhibit an *S. mutans* cultivated environment and reduce the degradation of collagen. Er:YAG laser irradiation improved the interaction between bioactive glass and dentin and enabled the formation of an intense crystalline layer, thereby promoting the enhancement of mineralization and avoiding daily erosion. We compared our proposed method with current commercial desensitizing systems, and the results suggested that our method is a durable treatment option for dentin hypersensitivity.

## Figures and Tables

**Table 1:** Percentage of dentinal tubule occlusion (%) and maximum penetration depth (µm) (*n* ≥ 20).

Dentin Specimens	Percentage of Dentinal Tubule Occlusion (%)	Maximum Penetration Depth (μm)
Group a	--	--
Group b	63 ± 30	4
Group c	94 ± 6	68
Group d	94 ± 6	>300

## Data Availability

Data is contained within the article.
